# Targeting RNA Polymerase I Transcription Activity in Osteosarcoma: Pre-Clinical Molecular and Animal Treatment Studies

**DOI:** 10.3390/biomedicines11041133

**Published:** 2023-04-09

**Authors:** Chang-Won Kang, Anneke C. Blackburn, Amos Hong Pheng Loh, Kuick Chick Hong, Jian Yuan Goh, Nadine Hein, Denis Drygin, Chris R. Parish, Ross D. Hannan, Katherine M. Hannan, Lucy A. Coupland

**Affiliations:** 1 The Division of Genome Science and Cancer, The John Curtin School of Medical Research, The Australian National University, Acton, Canberra 2601, Australia; chang-won.kang@anu.edu.au (C.-W.K.); kate.hannan@anu.edu.au (K.M.H.); 2 VIVA-KKH Paediatric Brain and Solid Tumour Programme, Children’s Blood and Cancer Centre, KK Women’s and Children’s Hospital, Singapore 229899, Singapore; 3 Department of Pathology and Laboratory Medicine, KK Women’s and Children’s Hospital, Singapore 229899, Singapore; 4 Regulus Therapeutics, 4224 Campus Point C, San Diego, CA 92121, USA; 5 Department of Biochemistry and Molecular Biology, University of Melbourne, Parkville 3010, Australia; 6 Department of Biochemistry and Molecular Biology, Monash University, Clayton 3800, Australia; 7 School of Biomedical Sciences, University of Queensland, St. Lucia 4067, Australia

**Keywords:** osteosarcoma, a small molecule inhibitor, RNA polymerase I transcription, CX-5461

## Abstract

The survival rate of patients with osteosarcoma (OS) has not improved over the last 30 years. Mutations in the genes *TP53*, *RB1* and *c-Myc* frequently occur in OS and enhance RNA Polymerase I (Pol I) activity, thus supporting uncontrolled cancer cell proliferation. We therefore hypothesised that Pol I inhibition may be an effective therapeutic strategy for this aggressive cancer. The Pol I inhibitor CX-5461 has demonstrated therapeutic efficacy in different cancers in pre-clinical and phase I clinical trials; thus, the effects were determined on ten human OS cell lines. Following characterisation using genome profiling and Western blotting, RNA Pol I activity, cell proliferation and cell cycle progression were evaluated in vitro, and the growth of *TP53* wild-type and mutant tumours was measured in a murine allograft model and in two human xenograft OS models. CX-5461 treatment resulted in reduced ribosomal DNA (rDNA) transcription and Growth 2 (G2)-phase cell cycle arrest in all OS cell lines. Additionally, tumour growth in all allograft and xenograft OS models was effectively suppressed without apparent toxicity. Our study demonstrates the efficacy of Pol I inhibition against OS with varying genetic alterations. This study provides pre-clinical evidence to support this novel therapeutic approach in OS.

## 1. Introduction

Osteosarcoma (OS) is the most frequently occurring primary malignant bone tumour in children and young adults, with the highest peak incidence during the second decade of life [1]. Currently, surgical resection with combination chemotherapy consisting of cisplatin, doxorubicin and high-dose methotrexate is used as the standard treatment for OS and has increased the 5-year overall survival rate to 60–70% in patients with localised disease [2]. However, 30% of OS patients do not respond to chemotherapy due to chemo-resistance, and the outcomes of OS patients with metastatic disease are lower than 20% at 5 years [3,4,5]. In addition, recent international clinical trials with more targeted therapies, such as trastuzumab in combination with methotrexate, doxorubicin and cisplatin (MAP) [6], MAP plus pegylated interferon alpha-2b (IFN- α-2b) [7], or MAP with ifosfamide and etoposide [8], in OS patients did not improve outcomes. Despite many studies, the overall survival rate in OS patients has plateaued for three decades; hence, new effective therapeutic treatments for improving OS outcomes are urgently needed.

Dysregulated cell growth is one of the representative hallmarks of cancer [9] and accompanies increased rates of protein synthesis and ribosome biogenesis in cancer cells [10]. RNA Polymerase I (Pol I) plays a role as a rate-limiting factor in ribosome biogenesis via transcription of the 47S precursor ribosomal RNA (47S rRNA), which is processed into mature 18S, 5.8S and 28S, the nucleic acid backbone of the ribosome [11,12,13]. In cancer cells, impaired signalling pathways (PI3K/Akt and Ras/MAPK) and genetic mutations in oncogenes (*c-Myc* and *mTOR*) and tumour suppressors (*TP53*, *RB1* and *ATRX*) promote Pol I transcription activity [12,14,15]. In combination, these findings led to the proposal that Pol I transcription activity suppression could be an effective therapy against cancers and to the development of new drugs inhibiting Pol I transcription.

CX-5461, the first-in-class small molecule inhibitor of Pol I-mediated transcription, suppresses rRNA transcription by preventing the binding of the Pol I-specific transcription initiation factor, SL-1, to the rDNA promoter region [16,17]. CX-5461 exhibits anti-tumour activity in the low nanomolar range with higher than 200-fold selectivity for Pol I transcription inhibition compared to Pol-II-mediated transcription [16,18]. Multiple pre-clinical studies have demonstrated the therapeutic efficacy of CX-5461 in several cancers, including B-cell lymphoma [18,19], acute myeloid leukaemia [11], multiple myeloma [20], prostate cancer [21], breast cancer [22], neuroblastoma [23] and ovarian cancer [24]. Furthermore, CX-5461 has demonstrated single-agent anti-tumour efficacy and was well tolerated in patients with haematological malignancies in phase I clinical trials. Palmar-plantar erythrodysesthesia was the only dose-limiting toxicity reported, and photosensitivity was a dose-independent adverse effect that could be managed by limiting sun exposure [17]. Other clinical trials of CX-5461 in solid tumours are ongoing (NCT02719977 and NCT04890613).

Recent studies in OS indicate that Pol I transcription activity is very likely to be dysregulated in OS. c-Myc protein, a key upstream regulator of ribosome biogenesis [25], is frequently overexpressed in OS [26], and the Pol I transcription-regulatory genes *TP53* and *RB1* are highly mutated [27,28,29,30]. Furthermore, enlarged nucleoli, a classic hallmark of elevated rDNA transcription activity, have also been described in human OS [31]. A previous study of RNA Pol I inhibition in osteosarcoma obtained relatively high 50% growth inhibitory concentration (GIC_50_) values for CX-5461 in two OS cell lines, and a high concentration of CX-5461 was used in cell cycle and autophagy studies [32]. These results provided preliminary evidence that inhibiting Pol I transcription using CX-5461 may be an effective therapy against OS. To provide greater justification for the clinical evaluation of CX-5461 in OS, this study aimed to undertake a comprehensive evaluation of the therapeutic efficacy of CX-5461 against OS. To do so, ten human OS cell lines were analysed for alterations in cancer-associated genes and the expression of key proteins involved in Pol I regulation. The concentrations of CX-5461 required to impact Pol I transcription, cell proliferation and cell cycle progression were determined. Additionally, the in vivo efficacy of CX-5461 was assessed in three murine models: (i) a *TP53* mutant human OS xenograft, (ii) a *TP53* wild-type human OS xenograft and (iii) a *TP53* wild-type murine allograft.

## 2. Materials and Methods

### 2.1. Compound

CX-5461 was purchased from SYNkinase (Parkville, Vic, Australia), and 10 mM stocks of CX-5461 were prepared in 50 mM NaH_2_PO_4_ (pH4.5) and diluted in growth media immediately prior to use in vitro. For the in vivo studies, 30 mg/kg of CX-5451 was prepared in 50 mM NaH_2_PO_4_ (pH4.5).

### 2.2. Cell Culture and Media 

Five commercially available human osteosarcoma cell lines, HOS, 143B, MNNG/HOS, U-2-OS and SJSA-1, were purchased from American Tissue Culture Collection (ATCC^®^, Manassas, VA, USA), and a further five patient-derived osteosarcoma cell lines, KOS-1, KOS-2, KOS-3, KOS-6 and KOS-7, were kindly provided by HS KIM from Seoul National University Hospital [33]. Human foreskin fibroblast cells (HF) were purchased from Lonza (Basel, Switzerland). HOS, 143B and MNNG/HOS cells were cultured with minimum essential medium (MEM) containing 10% foetal bovine serum (FBS) (Sigma-Aldrich, St Louis, MA, USA), 2 mM L-glutamine (Gibco, Waltham, MA, USA), 1 mM sodium pyruvate (Gibco, Waltham, MA, USA) and 1% Penicillin/Streptomycin/Neomycin (PSN) (Gibco, Waltham, MA, USA). U-2-OS cells were cultured with McCoy’s 5A medium (Gibco, Waltham, MA, USA) containing 10% FBS and 1% PSN. SJSA-1 cells were cultured with RPMI1640 containing 10% FBS, 1% PSN and 2 mg/mL of d-glucose (Gibco, Waltham, MA, USA). All five KOS cell lines were cultured with Dulbecco’s modified Eagle medium (DMEM) containing 10% FBS, 4 mM L-glutamine, 1% PSN and 1 mM sodium pyruvate. HF cells were cultured with DMEM containing 10% FBS, 8 mM L-glutamine and 1% PSN. All cells were cultured in a humidified cell culture incubator at 37 °C containing 5% CO_2_.

### 2.3. Genome Profiling of Cell Line

Single-nucleotide variants (SNVs) and copy number variants (CNVs) in ten OS cell lines were analysed using The Ampliseq^TM^ Cancer Childhood Panel DNA assay (Illumina, San Diego, CA, USA). The assay was conducted following the manufacturer’s instructions (Supplementary Methods).

### 2.4. IncuCyte-Based Cell Proliferation Assay

The proliferation of human OS cells (143B: 4.0 × 10^2^; HOS: 6.0 × 10^2^; MNNG/HOS: 6.0 × 10^2^; U-2-OS: 1.0 × 10^3^; SJSA-1: 1.0 × 10^3^; KOS-1: 1.0 × 10^3^; KOS-2: 1.0 × 10^3^; KOS-3: 1.0 × 10^3^; KOS-6: 1.0 × 10^3^; and KOS-7: 1.0 × 10^3^) and murine OS cells (2.0 × 10^3^) following exposure to fresh medium containing vehicle or various concentrations of CX-5461 for 72 h was undertaken as previously described [34]. The dose–response curve and the 50% growth inhibitory concentration (GIC_50_) were calculated using Graph-Pad Prism (version 9.1.0).

### 2.5. Western Blotting 

The protein levels of p53, MDM2, c-Myc and RB1 were assessed in the ten OS cell lines (1.0 × 10^6^) and human fibroblast cells (1.0 × 10^6^) as previously described [34] using primary and secondary antibodies (Supplementary Table S2) and β-actin as a loading control.

### 2.6. Quantitative Real-Time PCR for Assessment of rDNA Transcription

rDNA transcription in 1.0 × 10^5^ cells of all OS cell lines (HOS, 143B, MNNG/HOS, SJSA-1, U-2-OS, KOS-3, KOS-7 and K7M2) was estimated by quantitative real-time PCR (qRT-PCR) as previously described [19,34]. Briefly, cells were incubated with medium containing vehicle or various concentrations of CX-5461 for 1 h, following which total RNA was extracted and then quantified. RNA (500 ng) was treated with DNase and incubated in a T100^TM^ Thermocycler (Bio-Rad, Hercules, CA, USA), and then cDNA synthesis was performed with SuperScript^TM^ IV Reverse Transcriptase (Invitrogen, Carlsbad, CA, USA), Hexameric Random Primers (Promega, Madison, WI, USA) and dNTPs (Invitrogen, Carlsbad, CA, USA) in a T100^TM^ Thermocycler. All synthesised cDNA samples were loaded into 96-well PCR plates with SYBR^TM^ Green master mix, primers that targeted the external transcribed spacer-2 (ETS-2) of the pre-47S ribosomal RNA and the housekeeping gene, beta-2-microglobulin (β2M) (Supplementary Table S4), and real-time (RT) PCR was performed on a StepOne^TM^ Plus Real-Time PCR System (Applied Biosystems, Foster City, CA, USA). All settings for cDNA synthesis and RT-PCR are described in Supplementary Table S3. The dose–response curve and the 50% transcription inhibitory concentration (tIC_50_) were calculated using Graph-Pad Prism (version 9.1.0).

### 2.7. BrdU and PI Staining for Cell Cycle Analysis

All OS cell lines were analysed for cell cycle progression using Bromodeoxyuridine (BrdU) and Propidium iodide (PI) staining as previously described [34]. Briefly, OS cells were incubated for 72 h with medium containing vehicle or GIC_50_ CX-5461 concentrations (determined from the cell proliferation assay), then labelled with 10 µM BrdU (Sigma-Aldrich, St. Louis, MO, USA), harvested and stained with PI. Flow cytometry (Becton Dickinson LSR II) was used to assess the level of staining, and FlowJo software (version 10.0) was used for data analysis.

### 2.8. In Vivo Mouse Models

All in vivo experiments were conducted following approval from the Australian National University Animal Experimentation Ethics Committee (A2017/16 and A2020/36). Rag 2 knockout (KO) mice were obtained at 6 to 8 weeks of age from the Animal Resource Centre (Perth, Australia). 143B-Luc cells (1 × 10^6^) in 50 µL of Hank’s Balanced Salt Solution (HBSS) (Sigma-Aldrich, St Louis, MO, USA) or SJSA-1-Luc cells (5 × 10^6^) in 50 µL of HBSS containing 50% (*v*/*v*) Matrigel (Corning, NY, USA) were injected subcutaneously into the right flanks of mice. For the allograft mouse model, K7M2 cells (1 × 10^6^) in 50 µL of HBSS were injected subcutaneously into BALB/c wild-type mice on the right flank. Tumour-bearing mice were monitored daily. Once the average tumour volume reached 30~50 mm^3^, mice were randomised to receive either CX-5461 or vehicle. Thrice (3 times) weekly for 3 weeks, 30 mg/kg of CX-5461 in 50 mM NaH_2_PO_4_ (pH4.5) or the corresponding vehicle (50:50 mixture of 50 mM NaH_2_PO_4_ and 50 mM Citric Acid) was administered via oral gavage. The experiments were terminated 15 days following the start of drug administration or prior if mice reached the ethical endpoint.

### 2.9. Statistical Analysis

Statistical analyses were performed using GraphPad Prism software, version 9.1.0. The dose–response curve fit and 50% inhibitory values were obtained using non-linear regression. Comparisons of GIC_50_ and tIC_50_ between mutant p53 and wild-type p53 OS cell lines were conducted using a Mann–Whitney U test. Statistical significance between two groups (vehicle and CX-5461-treated groups) was assessed using two-way ANOVA. 

## 3. Results

### 3.1. CX-5461 Inhibits OS Cell Proliferation 

**Figure 1:**
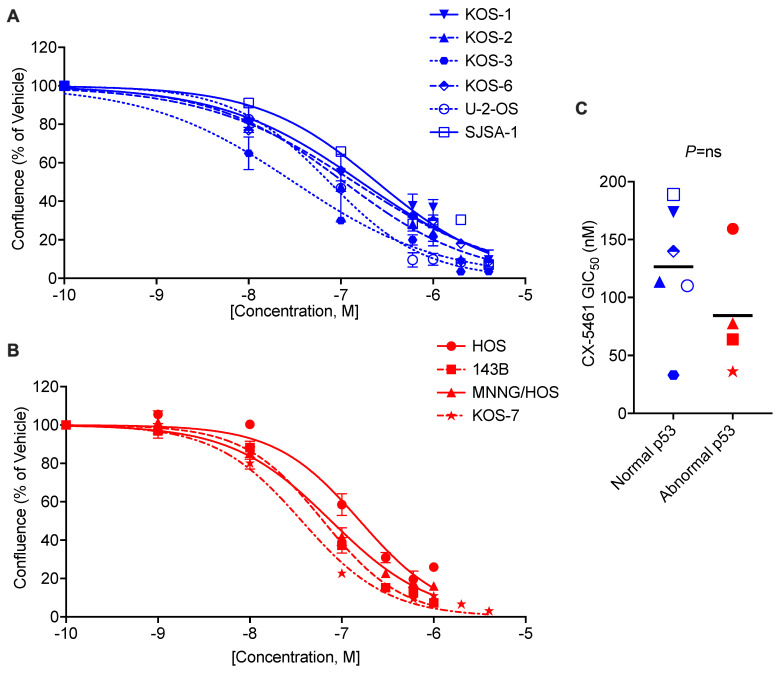
CX-5461 inhibits human OS cell proliferation irrespective of p53 status. (**A**) Normal p53 and (**B**) abnormal p53 osteosarcoma cell lines were treated with the indicated concentrations of CX-5461 or vehicle (50 mM NaH_2_PO_4_ (pH 4.5)) for 72 h and assessed for proliferation using the IncuCyte^®^ ZOOM Live Cell Imaging System. Dose–response curves and 50% growth inhibitory concentrations (GIC_50_) were calculated using non-linear regression analysis, mean +/− SEM of *n* = 3 biological replicates. (**C**) Effect of p53 status on GIC_50_ of CX-5461 with p53 status determined from genomic profiling data (Supplementary Table S1) and p53 protein expression levels in Western blots (Supplementary Figure S1). Normal p53: *TP53* wild type with the normal level of p53 protein expression; abnormal p53: *TP53* mutant or a dysregulated level of p53 protein expression. Each symbol represents the mean value of *n* = 3. Statistical analysis was conducted using a Mann–Whitney U test.

In our preliminary characterisation analysis using genome profiling and Western blotting, we confirmed that genes and proteins associated with RNA Pol I activity were mutated and dysregulated, respectively, in OS cell lines (Supplementary Figures S1 and S2 and Table S1), thus suggesting that CX-5461 may have anti-tumour effects on OS. Consistent with our expectations, CX-5461 exhibited a dose-dependent reduction in cell density (% of confluency) following 72 h of treatment (Figure 1A,B). Mutations in *TP53* are reported to confer chemo-resistance and a poor prognosis in OS patients [35]. CX-5461 effectively suppressed cell proliferation in both normal p53 (wild-type *TP53* gene and normal p53 protein expression) (Figure 1A) and abnormal p53 (mutated *TP53* gene or dysregulated p53 protein expression) (Figure 1B) OS cell lines with low nanomolar ranges of 50% GIC_50_ (*p53 abnormal:* HOS: 159 nM; 143B: 63 nM; MNNG/HOS: 77 nM; KOS-7: 36 nM; *p53 normal:* U-2-OS: 110 nM; SJSA-1: 188 nM; KOS-1: 173 nM; KOS-2: 113 nM; KOS-3: 33 nM; and KOS-6: 139 nM) (Figure 1C). There was a non-significant trend for normal p53 cell lines to have higher GIC_50_ values than abnormal p53 cell lines. These results suggest that CX-5461 has anti-tumour effects on both *TP53* mutant and wild-type OS tumours. 

### 3.2. CX-5461 Shows on-Target Activity in OS Cells

**Figure 2:**
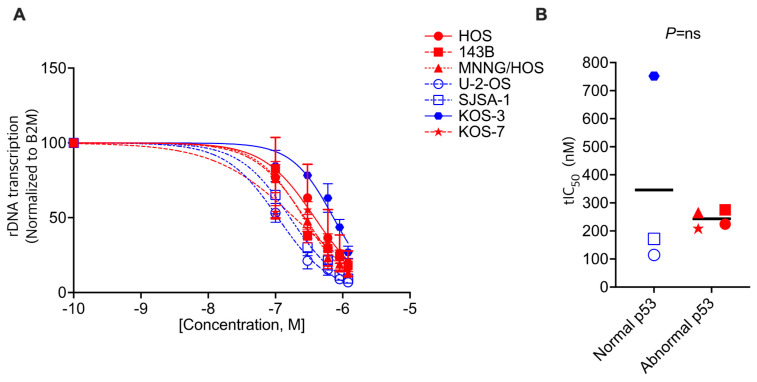
CX-5461 suppresses rDNA transcription in p53 normal and abnormal human OS cells. (**A**) ATCC and KOS (KOS-3 and KOS-7) osteosarcoma cell lines were treated with CX-5461 or vehicle (50 mM NaH_2_PO_4_ (pH4.5)) for 1 h, and the rDNA transcription rate was determined by qRT-PCR using the results of the external transcribed spacer (ETS) normalised to β2 microglobulin (β2M). The dose–response curve and tIC_50_ were estimated using non-linear regression analysis, mean +/− SEM of *n* = 3 biological replicates. (**B**) Effect of p53 status on tIC_50_ of CX-5461. Each symbol represents the mean value of *n* = 3. Statistical analysis was conducted using a Mann–Whitney U test.

CX-5461 was designed to inhibit Pol I transcription activity by disrupting the transcription initiation complex on the RNA Pol I promoter region, a fundamental component for Pol I transcription [12]. The on-target activity and mechanism of action of CX-5461 have been demonstrated in human cancers in pre-clinical and clinical studies [16,17]. To estimate the on-target activity of CX-5461 on rDNA transcription in OS cell lines, the abundance of a rapidly processed region of the 47S pre-ribosomal RNA [12] was investigated using qRT-PCR with CX-5461 treatment. The rDNA transcription rate was found to be reduced by CX-5461 in a dose-dependent manner within 1 h of treatment in all OS cell lines tested (Figure 2A). Except for in the KOS-3 cell line, CX-5461 showed a similar 50% transcription inhibitory concentration (tIC_50_) range in normal p53 (U-2-OS: 114 nM; SJSA-1: 171 nM; KOS-3: 752 nM) and abnormal p53 OS cell lines (HOS: 221 nM; 143B: 275 nM; MNNG/HOS: 265 nM; KOS-7: 207 nM) (Figure 2B). In the genomic study of KOS-3, no mutations in genes associated with Pol I transcription regulation were detected, which may explain the relative resistance to Pol I inhibition. The results in this section demonstrate that CX-5461 effectively inhibits its primary target, RNA Pol I, regardless of p53 status.

### 3.3. CX-5461 Leads to G2-Phase Cell Cycle Arrest

**Figure 3:**
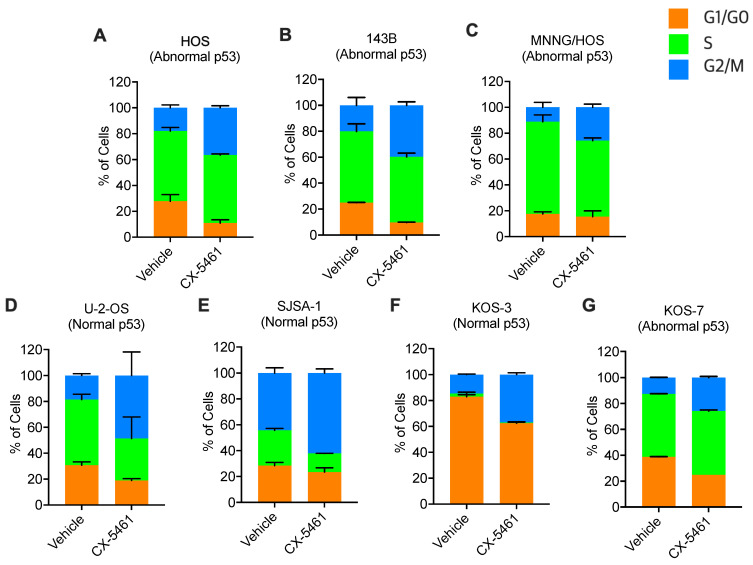
CX-5461 induces G2-phase cell cycle arrest in human OS cells. (**A**–**G**) ATCC and KOS osteosarcoma cell lines were treated with the respective GIC_50_ CX-5461 dose (determined from Figure 2) or vehicle (50 mM NaH_2_PO_4_ (pH4.5)) for 72 h, and the proportion of cells in each cell cycle phase was determined using BrdU/PI staining and flow cytometric analysis. Mean +/− SD of *n* = 2 biological replicates. G1/G0 = quiescent and growth phases not involving proliferation; S = DNA synthesis phase; and G2/M = growth and preparation for mitosis and mitosis phases.

HOS, 143B, MNNS/HOS and KOS-7 were identified as abnormal p53 (p53 mutant) cell lines, and U-2-OS, SJSA-1 and KOS-3 were identified as normal p53 (p53 wild-type) cell lines (Supplementary Figure S1 and Table S1). Previously, it has been reported that CX-5461 treatment leads to varying consequences in cell cycle progression that correlate with p53 status: (i) p53 wild-type cancers: G1-phase cell cycle arrest [19,36]; (ii) p53 mutant cancers: G2-phase cell cycle arrest [36]. To determine the effects of CX-5461 on OS cell cycle progression, a BrdU and PI staining assay was performed. The HOS, 143B, MNNG/HOS, U-2-OS, SJSA-1, KOS-3 and KOS-7 cell lines were treated with the GIC_50_ of CX-5461 for 72 h and analysed by flow cytometry. CX-5461 treatment was found to induce G2-phase cell cycle arrest in all OS cell lines analysed, both p53 normal and abnormal cell lines (Figure 3). 

### 3.4. CX-5461 Reduces Tumour Growth in Human OS Xenografts 

**Figure 4:**
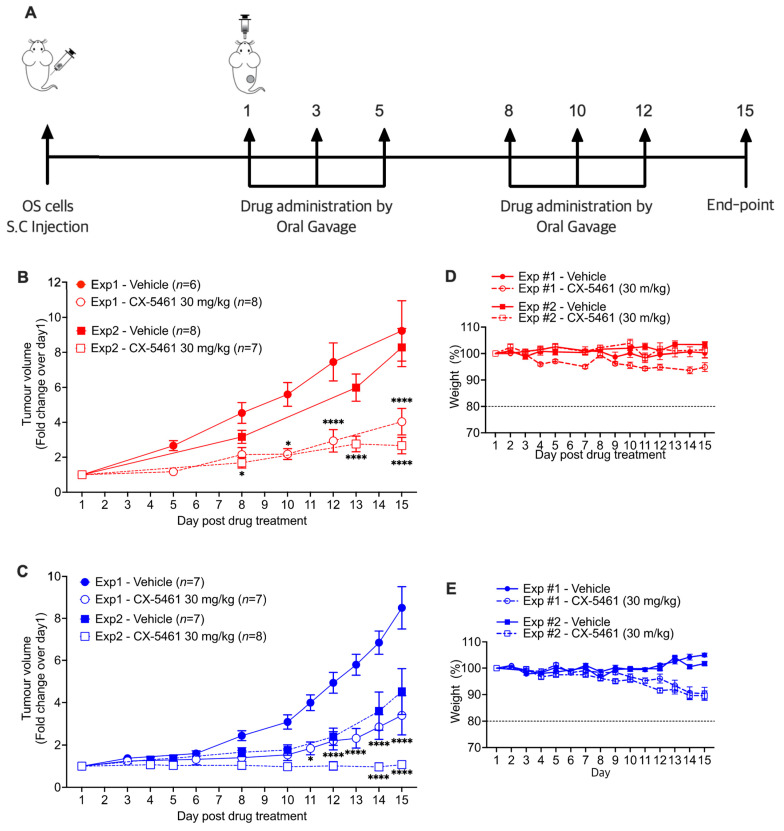
CX-5461 reduces tumour growth in human OS xenograft mouse models. (**A**) Schematic of human OS xenograft mouse model: OS cells were injected subcutaneously into the right flanks of male and female Rag2 KO mice; then, when tumours could be measured using digital callipers, mice were randomised (ensuring equal distribution of sex and tumour size) to vehicle (50 mM NaH_2_PO_4_ (pH4.5)) or CX-5461 (30 mg/kg) group thrice weekly for two weeks. (**B**) 143B-Luc OS cells (abnormal p53; 1 × 10^6^ cells in 50 µL of HBSS) or (**C**) SJSA-1-Luc OS cells (normal p53; 3 × 10^6^ in 50 µL of HBSS containing 50% Matrigel) were injected. Tumour volume was monitored and expressed in each mouse as the fold change in the tumour size at the start of treatment, with the average of each treatment group depicted. (**D**,**E**) The weight of each mouse was monitored during the treatment period, with a 20% reduction in body weight being the ethical end point (dotted line), which was not reached. Mean +/− SEM of biological replicates as indicated. Statistical analysis was performed using two-way ANOVA, * p < 0.05, **** p < 0.0001.

The anti-tumour activity of CX-5461 was investigated in two human OS xenograft mouse models in Rag 2 knockout (KO) mice using 143B (p53 mutant-type) and SJSA-1 (p53 wild-type) cell lines (Figure 4). Each experiment was repeated to demonstrate the reproducibility of the results. CX-5461 effectively suppressed 143B tumour growth, and this effect was significant (*p* < 0.05) after the first week (Day 8) of drug dosing (Figure 4B). Similarly, CX-5461 showed tumour-growth-inhibitory activity on SJSA-1 tumours, and the effect was significant on Day 11 of treatment (Figure 4C). CX-5461 was well tolerated without excessive reductions in body weight (Figure 4D,E). 

### 3.5. CX-5461 Demonstrates Therapeutic Efficacy in an Immunocompetent Mouse Model of OS

**Figure 5:**
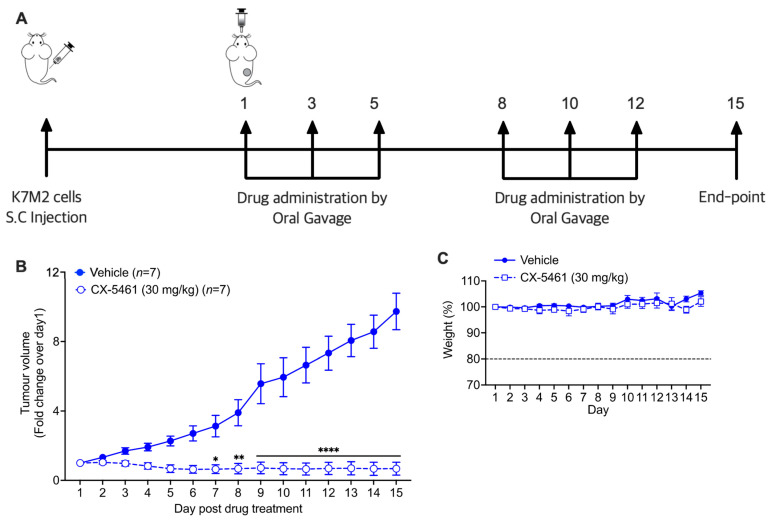
CX-5461 inhibits tumour cell growth in an immune-competent mouse model of osteosarcoma. (**A**) Schematic of immune-competent murine model of osteosarcoma: K7M2 murine OS cells (1 × 10^6^ in 50 µL of HBSS) were injected subcutaneously into the right flanks of male and female BALB/c mice; then, when tumours could be measured using digital callipers, mice were randomised (ensuring equal distribution of sex and tumour size) to vehicle (50 mM NaH_2_PO_4_ (pH4.5)) or CX-5461 (30 mg/kg) group thrice weekly for two weeks. (**B**) Tumour volume was monitored and expressed in each mouse as the fold-change in the tumour size at the start of treatment, with the average of each treatment group depicted. (**C**) The weight of each mouse was monitored during the treatment period, with a 20% reduction in body weight being the ethical end point (dotted line), which was not reached. Mean +/− SEM of biological replicates as indicated. Statistical analysis was performed using two-way ANOVA; * p < 0.05, ** p < 0.005, **** p < 0.0001.

To investigate whether CX-5461 had the same in vivo therapeutic efficacy in the presence of a functional immune system, the anti-tumour effect of CX-5461 was evaluated in an allograft mouse model using the murine OS cell line K7M2 (Figure 5). As seen in the human OS xenograft mouse models, CX-5461 effectively suppressed K7M2 tumour growth in BALB/c mice and was well tolerated without significant changes in animal body weight (Figure 5B,C). The in vitro studies of CX-5461 on the K7M2 cell line demonstrated the suppression of RNA Pol I transcription and cell proliferation via G2-phase cell cycle arrest (Supplementary Figure S3).

## 4. Discussion

The advancement of diagnostic and therapeutic strategies for OS has not improved outcomes over the last 30 years, and chemo-resistance is a major problem. There is, therefore, an urgent need for new therapeutic strategies against this disease [1,7,8,37]. Accelerated RNA Pol I transcriptional activity is common to many cancers, including OS; thus, targeting this protein represents a novel therapeutic approach. In this study, we have (i) determined the CX-5461 GIC_50_ and tIC_50_ values for each OS cell line, (ii) demonstrated the on-target effect of CX-5461 on Pol I activity, (iii) analysed the effect of CX-5461 on cell division in a range of cell lines and (iv) demonstrated the therapeutic effect of CX-5461 in vivo using three different mouse models. 

Alterations in tumour suppressors and oncogenes that occur in many cancers influence Pol I transcription activity [12,14]. The tumour suppressors p53 and RB1 suppress Pol I transcription by blocking the assembly of the pre-initiation complex, SL-1 and UBF, on the rDNA promoter region [38,39]. p53 and RB1 also suppress the transcription of the oncogene c-Myc, which enhances Pol I transcription via stimulation of UBF and SL-1 binding to the rDNA promoter region [25,40,41]. Our preliminary characterisation analysis of the OS cell lines identified molecular alterations that would impact the presence of mutations in *TP53*, *RB1*, *TSC1*, *TSC2* and *ATRX* and amplifications of *c-Myc*, *MDM2* and *CDK4*. Additionally, the dysregulated expression of p53, RB1, c-Myc and MDM2 proteins was demonstrated, all of which have the potential to impact Pol I activity. These molecular results are consistent with other genetic studies of OS and highlight the significant diversity in gene and protein alterations that may lead to OS and enhanced Pol I activity, and they emphasise the potential for Pol I inhibitors to be effective against the majority of OS cases.

Multiple studies have revealed the mechanism of the therapeutic effect of CX-5461 on human cancers [11,16,17,18,20,24,36,42,43]. CX-5461 prevents the assembly of the Pol I-specific pre-initiation complex factor, SL-1, on the rDNA promoter region, leading to the abrogation of Pol I binding [18]. Consequently, these changes result in anti-tumour effects through the p53-dependent nucleolar stress response (NSR) and p53-independent NSR pathways [19,36,44]. In this study, CX-5461 demonstrated anti-cancer activity across the panel of 10 OS cell lines. CX-5461 was shown to rapidly suppress rDNA transcription, the primary target, within 1 h of treatment, and subsequently induced anti-proliferative effects in all OS cell lines, both normal p53 and abnormal p53, through G2-phase cell cycle arrest. These results extend the evidence of CX-5461’s anti-tumour effects on OS, irrespective of the p53 status, thus implying the same multi-pathway mechanisms.

In clinical studies, the response to chemotherapy and the survival rate of OS patients varied according to the *TP53* gene mutation status [45,46]. A meta-analysis of eight published studies demonstrated that patients with tumours bearing *TP53* mutations had a significantly higher risk of death within 2 years than patients with wild-type *TP53* tumours [35]. Our in vivo efficacy studies of CX-5461 showed a significant and reproducible therapeutic effect on both p53 mutant OS, 143B, and p53 wild-type OS, SJSA-1, tumours. CX-5461 also exhibited a significant tumour-growth-inhibitory effect in an allograft mouse model with a functional immune system. A previous study of CX-5461 on human OS in vitro found that CX-5461 induced anti-tumour activity via the activation of autophagy involving the mammalian target of rapamycin (mTOR) signalling pathways in the OS cell lines U-2-OS and MNNG/HOS [32]. The drug doses used in this study were significantly higher (43-fold) than those used in our studies. CX-5461 was also observed to suppress U-2-OS tumour growth in an in vivo mouse model.

Previous studies of the anti-proliferative effect of CX-5461 in 50 cancer cell lines and 5 normal cell lines demonstrated a median GIC_50_ value in the cancer cell lines of 147 nM (range 3–5500 nM), whereas the 5 normal cell lines all had a GIC_50_ value of approximately 5000 nM [16]. The GIC_50_ values obtained for the 10 OS cell lines in this study (33–188 nM), therefore, closely align with the median values previously reported for other cancer cell lines. More importantly, these values are well below those required to impact the proliferation of normal cell lines; thus, these doses could be considered selective for OS cells. The lack of obvious side effects in our mouse studies, with a maximum weight loss of 10% measured, reflects the results of early phase trials in humans in which CX-5461 was well tolerated [17]. Interestingly, the most profound anti-tumour effect of CX-5461 was seen in the immune-competent mouse model, which also displayed the most rapid tumour growth and the least weight loss. As immune cells may enhance or inhibit tumour growth, further studies would be required to determine what effects CX-5461 has on the cells of the immune system in this model.

As with most chemotherapy agents, resistance to RNA Pol I inhibition may develop as a result of new mutations or compensatory mechanisms. Combining therapies that target different pathways increases the treatment effect and decreases the likelihood of resistance occurring. CX-5461 was successfully combined with the mTORC1 inhibitor everolimus to demonstrate a synergistic anti-tumour effect in lymphoma-bearing mice [17]. Future trials in OS could combine CX-5461 with more commonly used agents, such as methotrexate, doxorubicin and cisplatin.

In conclusion, this study has identified mutations in genes associated with Pol I activity in 10 OS cell lines and has confirmed the alteration of proteins well recognised to be involved in the regulation of Pol I transcription. Furthermore, this study has demonstrated that targeting elevated Pol I transcription in OS shows significant promise as an effective novel therapeutic strategy. Given the pre-clinical results provided and the safety profile of CX-5461 already obtained in human clinical studies, CX-5461 should be considered a potential therapeutic option for OS.

## Data Availability

The data presented in this study are available on request from the corresponding author.

## Supplementary Materials

The following supporting information can be downloaded at: https://www.mdpi.com/article/10.3390/biomedicines11041133/s1, Figure S1: Western blots of four key proteins that are known regulator of RNA Pol I activity; Figure S2: Uneditied western blots of four key proteins that are known regulator of RNA Pol I activity; Figure S3: In vitro antitumour effects of CX-5461 on K7M2; Table S1: Genetic mutations in OS cell lines; Table S2: List of antibodies; Table S3: qPCR setting; Table S4: qPCR primer sequences.

## Abbreviations

Akt	AKT serine/threonine kinase 1
ATM	Ataxia telangiectasia-mutated kinase
ATR	Ataxia telangiectasia and Rad3-related kinase
ATRX	Alpha-thalassemia/mental retardation x-linked chromatin remodeller
CDK4	Cyclin-dependent kinase 4
c-Myc	Cellular myelocytomatosis
GIC_50_	50% growth inhibitory concentration
KO	Knockout
MAP	Methotrexate, doxorubicin and cisplatin
MAPK	Mitogen-Activated Protein Kinase
MDM2	E3 ubiquitin-protein ligase MDM2
mTOR	Mammalian target of rapamycin
OS	Osteosarcoma
p53	Tumour suppressor protein 53
Pol I	RNA Polymerase I
PI3K	Phosphoinositide 3-kinase
rDNA	Ribosomal DNA
RB1	Retinoblastoma 1
SL-1	Selective factor 1
tIC_50_	50% transcription inhibitory concentration
*TP53*	p53 gene
TSC	Tuberous Sclerosis Complex
UBF	Upstream binding factor
