# Supplementary material for: Targeting RNA Polymerase I Transcription Activity in Osteosarcoma: Pre-Clinical Molecular and Animal Treatment Studies
Source: Biomedicines. 2023 Apr 9;11(4):1133. doi: 10.3390/biomedicines11041133 (PMC10135452; doi:10.3390/biomedicines11041133)
Supplement: Supplementary file 1 [file biomedicines-11-01133-s001.zip › biomedicines-2307131-supplementary.pdf]

Supplementary Data

1. Supplementary methods

Genome profiling assay

The Ampliseq™ Cancer Childhood Panel DNA assay (Illumina) detects single nucleotide variants (SNV) from hotspots of 86 genes, full exons of 44 genes, and copy number variants (CNV) from 28 clinically relevant cancer genes. DNA was extracted from the cell lines using the ReliaPrep tissue DNA extraction kit (Promega, Madison, WI, USA). The Childhood Cancer Research Assay primers and AmpliSeq Library Kit Plus (Illumina) which were used for library preparation. The prepared libraries were sequenced using a MiniSeq sequencer (Illumina). Base calling and mapping to a reference genome (hg19) was performed using the BaseSpace Informatics suite (Illumina). SNV variant calling was performed in DNA amplicon application (Illumina, version 2.1.1). CNV calling was performed in OncoCNV caller application (Illumina, version 1.2.0). All VCF files were loaded into variant interpreter (version 2.7.0.412) for interpretation. SNV somatic candidates were selected based on minimum coverage reads of more than 100, minimum allele frequency of 10%, cosmic reported variant and less than 1% prevalence in the 1000 Genome Population Database. CNV candidates with more than 10 gene copies were identified.

2. *Supplementary figures*

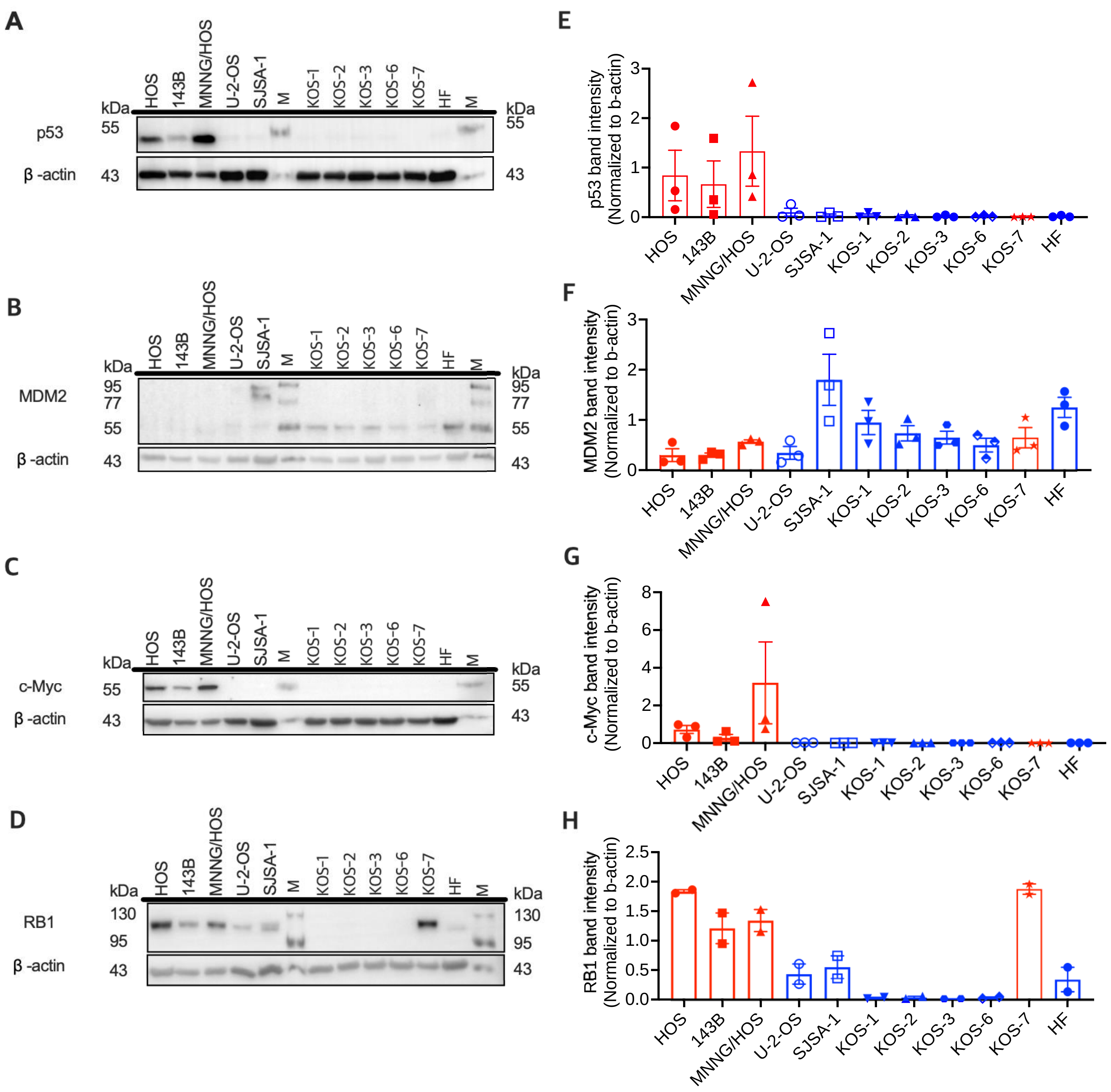

Supplementary Figure S1. Four key proteins that are known regulators of RNA Pol I activity are dysregulated in human OS cell lines.

Basal levels of **A** p53, **B** MDM2, **C** c-Myc and **D** RB1 protein expression were evaluated by western blot analysis with β-actin used as an internal loading control. **E–H**, Quantification of band intensities are shown following normalisation to β-actin. The blots shown are representative of independent biological replicates (p53, MDM2 and c-Myc: *n*=3, RB1: *n*=2). Colours indicate p53 status determined from sequencing and western blot analysis (Supplementary Table 1). Normal p53: *TP53* wild type

with the normal level of p53 protein expression, Abnormal p53: *TP53* mutant or the dysregulated level of p53 protein expression. M= molecular marker; HF = human fibroblasts.

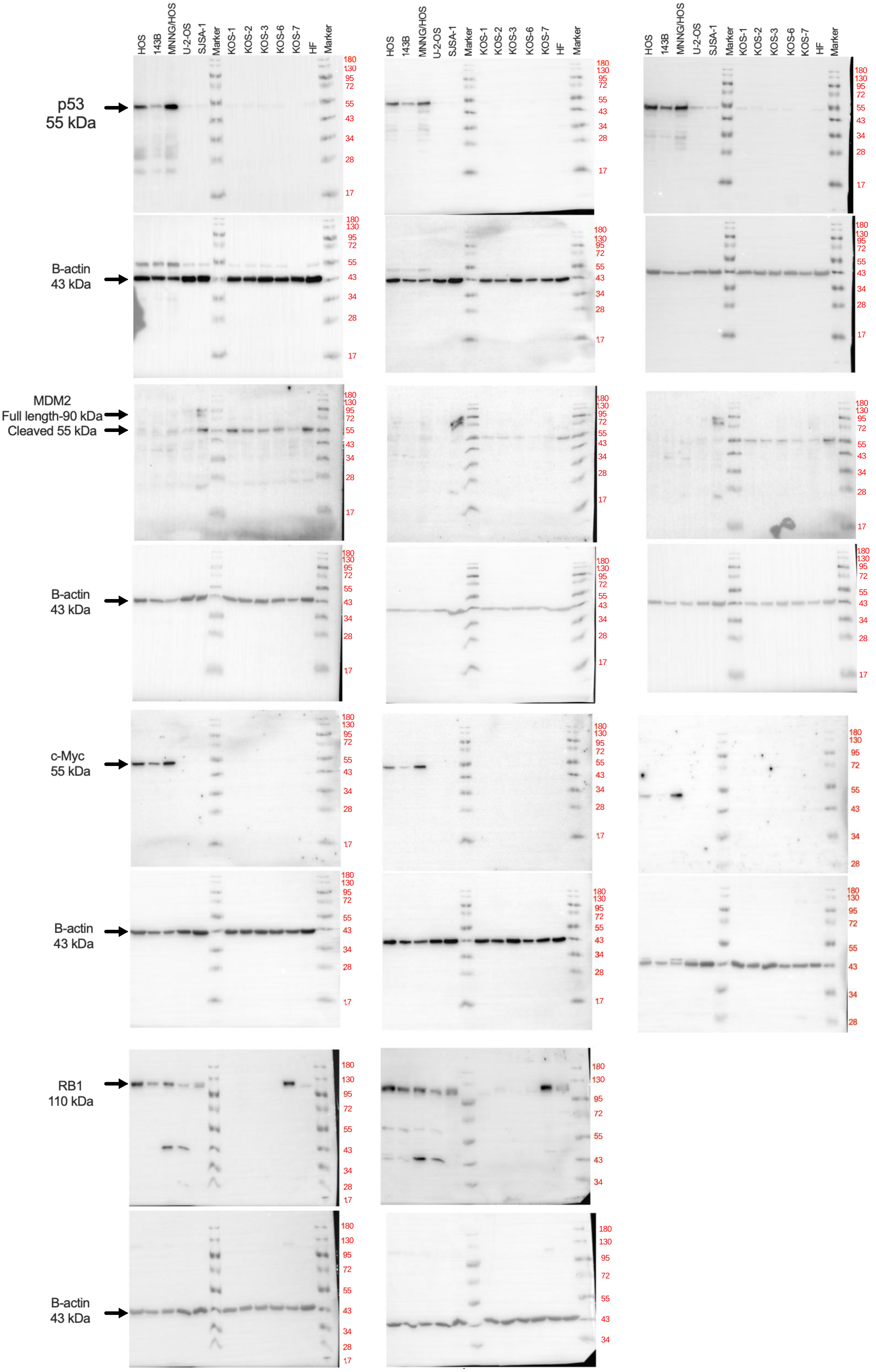
HOS 143B MNNG/HOS U-2-OS SJSA-1 Marker KOS-1 KOS-2 KOS-3 KOS-6 KOS-7 HF Marker
HOS 143B MNNG/HOS U-2-OS SJSA-1 Marker KOS-1 KOS-2 KOS-3 KOS-6 KOS-7 HF Marker
HOS 143B MNNG/HOS U-2-OS SJSA-1 Marker KOS-1 KOS-2 KOS-3 KOS-6 KOS-7 HF Marker
p53
55 kDa
B-actin
43 kDa
MDM2
Full length-90 kDa
Cleaved 55 kDa
B-actin
43 kDa
c-Myc
55 kDa
B-actin
43 kDa
RB1
110 kDa
B-actin
43 kDa
180 130 95 72 55 43 34 28 17

Supplementary Figure S2. Unedited western blots of four key proteins that are known regulators of RNA Pol I activity in OS cell lines. p53, MDM2, c-Myc and RB1 protein expression were estimated by western blot analysis.

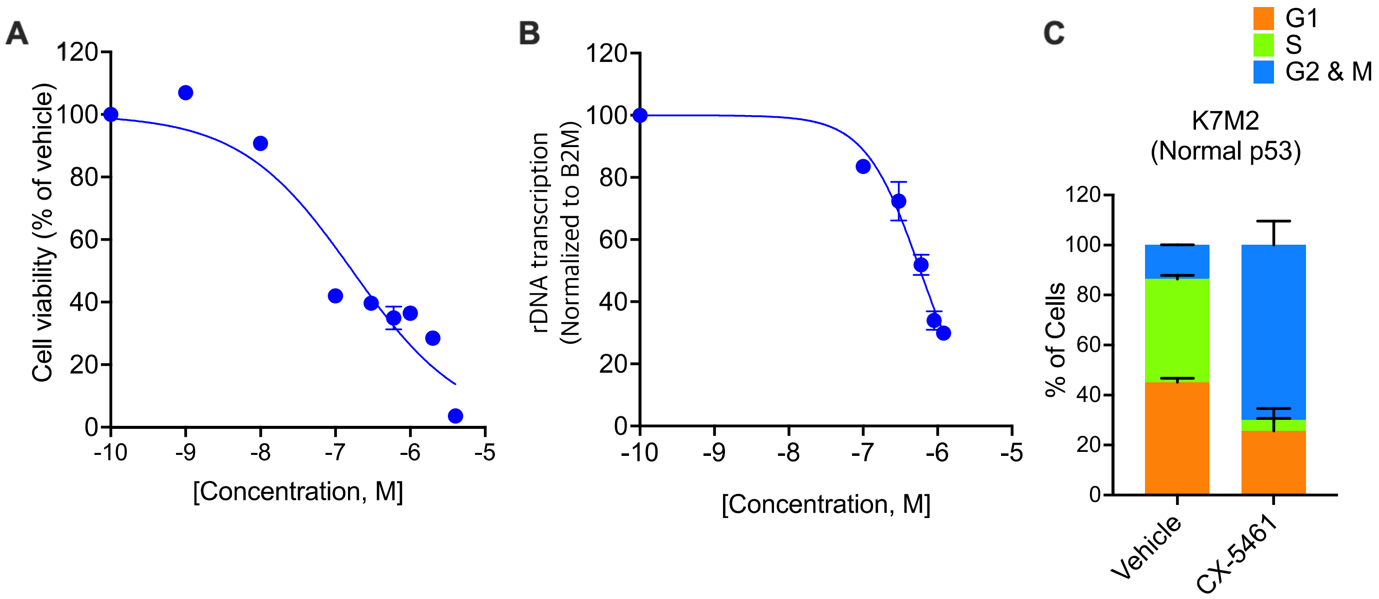

Supplementary Figure S3. CX-5461 shows anti-tumour effects on murine OS cell line, K7M2, in-vitro

A Murine OS K7M2 cells were treated with various concentrations of CX-5461 or vehicle for 72 hours and assessed for proliferation via IncuCyte® ZOOM Live Cell Imaging System. A dose-response curve with GIC_{50} of CX-5461 was generated using non-regression analysis with mean +/- SEM of *n*=3 biological replicates. **B** The effects of CX-5461 or vehicle for 1 hour on rDNA transcription activity in K7M2 cells was estimated by qRT-PCR with the result of the external transcribed spacer (ETS) normalised to β2 microglobulin (β2M). A dose-response curve was calculated using non-linear regression analysis, mean +/- SEM of *n*=3 biological triplicates. **C** K7M2 cells treated with the GIC_{50} CX-5461 dose (585 nM) determined from (**A**) or vehicle for 72 hr and cell cycle progression was analysed using BrdU/PI staining and flow cytometry, mean +/- SD of *n*=2 biological duplicates.

3. Supplementary Tables

Supplementary Table S1. Genetic mutations in OS cell lines.

Cell line	Genes	Names	CNVs*	Mutation status	Consequence
	Tumour suppressors				
HOS	TP53	Tumour protein p53		p.Arg156Pro	Missense variant
	PTCH1	Protein patched homolog 1		p.Arg1442Trp	Missense variant
	TSC1	Tuberous sclerosis-1		c.2626-4dupT	Splice region variant, Intron variant
	TSC2	Tuberous sclerosis-2		p.Arg1706Cys	Missense variant
	FASLG	Tumour Necrosis Factor Ligand superfamily member 6		p.Phe87Leu	Missense variant
	ARID1B	AT-rich interaction domain 1B		p.Gln131dup	Inframe insertion
	NF1	Neurofibromin 1		p.Val689Met	Missense variant
	TCF3	Transcription factor 3		p.Gly431Ser	Missense variant
	Tumour suppressors				
143B	TP53	Tumour protein p53		p.Arg156Pro	Missense variant
	PTCH1	Protein patched homolog 1		p.Arg1442Trp	Missense variant
	TSC1	Tuberous sclerosis-1		c.2626-4dup	Splice region variant, Intron variant
	TSC2	Tuberous sclerosis-2		p.Arg1706Cys	Missense variant
	FASLG	Tumour Necrosis Factor Ligand superfamily member 6		p.Phe87Leu	Missense variant
	ARID1B	AT-rich interaction domain 1B		p.Gln131dup	Inframe insertion
	NF1	Neurofibromin 1		p.Val689Met	Missense variant
	TCF3	Transcription factor 3		p.Gly431Ser	Missense variant
	Tumour suppressors				
MNNG/HOS	RB1	Retinoblastoma 1		c.1696-3T>C	Splice region variant, Intron variant

	TP53	Tumour protein p53	p.Arg156Pro	Missense variant
	PTCH1	Protein patched homolog 1	P.Arg1442Trp	Missense variant
	TSC1	Tuberous sclerosis-1	c.2626-4dupT	Splice region variant, Intron variant
			c.2626-5_2626-4dupTT	Splice region variant, Intron variant
	TSC2	Tuberous sclerosis-2	p.Arg1706Cys	Missense variant
	FASLG	Tumour Necrosis Factor Ligand superfamily member 6	p.Phe87Leu	Missense variant
	ARID1B	AT-rich interaction domain 1B	p.Gln131dup	Inframe insertion
	NF1	Neurofibromatosis type 1	p.Val689Met	Missense variant
	TCF3	Transcription factor 3	p.Gly431Ser	Missense variant
	Tumour suppressors			
U-2-OS	TET2	Methylcytosine dioxygenase TET2		
	TSC1	Tuberous sclerosis-1	c.2626-5_2626-4dupTT	Splice region variant, Intron variant
			c.2626-4dupT	Splice region variant, Intron variant
	TSC2	Tuberous sclerosis-2	p.Val1144Met	Missense variant
	Oncogenes			
	H3F3A	H3 histone, family 3A	p.Val36Leu	Missense variant
			p.Gly35_Val36insPhe	Inframe insertion
	PPM1D	Protein Phosphatase 1D	p.Arg458Ter	Stop gained
	Tumour suppressors			
SJSA-1	TSC2	Tuberous sclerosis-2	p.Pro1305Leu	Missense variant
	ARID1B	AT-rich interaction domain 1B	p.Gly953Ser	Missense variant
			p.Gln131dup	Inframe insertion
	KMT2D	Histone-lysine N-methyltransferase 2D	p.Tyr2199IlefsTer65	Frameshift variant

				p.Tyr2196Ter	Stop gained
	TCF3	Transcription factor 3		p.Gly1196Ser	Missense variant
	Oncogenes				
	CDK4	Cyclin-dependent kinase 4	40		
	NRAS	GTPase NRas		p.Gln61Lys	Missense variant
	MET	Tyrosine-protein kinase Met		p.Met362Thr	Missense variant
	ERBB3	Receptor tyrosine-protein kinase erbB-3		p.Glu173Gln	Missense variant
	MDM2	E3 ubiquitin-protein ligase Mdm 2	66.5	p.Glu333Asp	Missense variant
	RHOA	Ras homolog family member A		p.Val43Ala	Missense variant
	GLI1	Glioma-associated oncogene homolog 1	40		
	Tumour suppressors				
KOS-1	TSC2	Tuberous sclerosis-2		p.Met286Val	Missense variant
	NF1	Neurofibromatosis type 1		p.Met645Val	Missense variant
	NF2	Neurofibromatosis type 2		p.Thr480Met	Missense variant
	CREBBP	CREB-binding protein		p.Leu551Ile	Missense variant
	Tumour suppressors				
KOS-2	ASXL2	Additional sex combs-like protein 2		p.Gly1134Ser	Missense variant
	Tumour suppressors				
KOS-3	KMT2D	Histone-lysine N-methyltransferase 2D		p.Met3349Val	Missense variant
	CIC	Capicua transcriptional repressor		c.7187-8A>G	Splice region variant, Intron variant
	Tumour suppressors				
KOS-6	TSC1	Tuberous sclerosis-1		p.Pro613Ser	Missense variant
	CEBPA	CCAAT/enhancer binding protein alpha		p.His195_Pro196dup	Inframe insertion
	ARID1B	AT-rich interaction domain 1B		p.Pro605Ser	Missense variant
	Tumour suppressors				

KOS-7	TSC1	Tuberous sclerosis-1		p.Pro613Ser	Missense variant
	ATRX	Alpha thalassemia/mental retardation syndrome X-linked protein		p.Asp412Gly	Missense variant
	PDGFR	Platelet derived growth factor receptor alpha	9		
	Oncogene				
	MYC	Myelocytomatosis	11.5		

***CNVs (Copy Number Variants): > 10 gene copies identified.**

Supplementary Table S2 – Antibodies Used

**Primary Antibodies**			
**Human Target Protein**	**Raised in**	**Company**	**Catalogue number**
p53	Mouse	Santa Cruz, Dallas, TX	SC-126
RB1	Rabbit	Santa Cruz, Dallas, TX	SC-50
c-Myc	Mouse	Calbiochem, San Dieago, CA	OP10L
MDM2			
β-actin	Rabbit	Cell Signaling Technology, Danvers, MA	4970S
**Secondary Antibodies**			
**Target Protein**	**Raised in**	**Company**	**Catalogue number**
Rabbit IgG (HRP conjugated)	Goat	Bio-Rad, Hercules, CA	1706515
Mouse IgG (HRP conjugated)	Goat	Bio-Rad, Hercules, CA	1706516

Supplementary Table S3 – qPCR Settings Used

	**Time and Temperature setting**
**DNase treatment**	37° C for 15 min -> 70° C for 15 min -> 4° C
**cDNA synthesis**	20° C for 5 min -> 37° C for 5 min -> 42° C for 75 min -> 70° C for 15 min -> 4° C -> Store at -20° C
**RT-PCR**	Holding stage: 95° C for 20 sec<br>Cycling Stage (40 cycles): 95° C for 3 sec -> 60° C for 30 sec<br>Melt curve stage (Melt curve 0.7): 95° C for 15 sec -> 60° C for 1 min -> 95° C for 15 sec

Supplementary Table S4 – qPCR Primer Sequences

**Name of primer**	**Sequence**
ETS-2 forward	5′-GGCGGTTTGAGTGAGACGAGA-3′
ETS-2 reverse	5′-ACGTGCGCTCACCGAGAGCAG-3′
β2M forward	5′-CCGTGGVVTTAGCTGTGCTCGC-3′
β2M reverse	5′-CCCACTTAACTATCTTGGGCTG-3′
